# Notes on Iron, Etc.

**Published:** 1891-01

**Authors:** B. F. Church

**Affiliations:** Austin


### Items of Interest.

[CULLED BY DR. B. F. CHURCH, AUSTIN.]

**Some Notes Bearing on the Administration of Iron**, by John Aulde, M. D., Philadelphia, Pa.—Although iron is highly esteemed as a medicament, and is largely used for its tonic effect upon the system, so frequently does it occur that the patient objects, owing to some idiosyncrasy or fancy, that we cannot regard it wholly as an ideal hæmatinic. No apology, therefore, is required in offering to the profession a comparatively recent preparation, which is free from some of the objections that have been urged against many of the iron preparations now in use. In order to make the reason which I have to offer clear and distinct to the casual reader, I have deemed it wise to consider briefly some points intimately connected with the pharmacology of the drug. From this preliminary study we shall be in a measure prepared to estimate how nearly the new product comes to meeting the defects with which we have had to contend so long, and at the same time it may possibly lead to a more intelligent use or this well-known remedy.

Besides the reduced iron, we have in general use the ferric and ferrous preparations, the latter being more mild, less astrigent, and free from the objections to the ferric salts—that of coagulating albumin. Lethal doses of the ferric salts used intravenously, in experimental investigations, cause almost immediate paralysis of the central nervous system, fall of blood-pressure, and death. Although the perchloride, when thus used, causes instant death by coagulation of the blood, it does not act in this direct manner when introduced subcutaneously; the nerves are unaffected, but at the points of elimination inflammatory action is set up, *e. g.*, the kidneys, liver, and intestinal membrane show more or less effect.

Absorption takes place as a peptonate or albuminate, but it is taken up so slowly that no appreciable result follows, unless, as just stated, it may be used intravenously or subcutaneously.

Absorption takes place more rapidly in catarrhal conditions of the intestinal tract —a fact to be borne in mind when exhibiting large doses, which cause gastro-intestinal catarrh. Small doses do not have this effect, nor does the metal appear in the urine from their administration, such as may be observed after the ingestion of large doses. It will be inferred from the foregoing that by the exhibition of small doses of a soluble preparation of iron it will be assimilated without causing derangement of the alimentary tract, and in this way the secondary effects, *i. e.*, the deposit of the metal in the system, may be avoided.

The fact should be kept constantly in view, that metals have a poisonous action upon nerves, nerve-centres, and upon all glandular structures; and as iron is a reputed hæmatinic, much harm may result from its injudicious employment, as there are evidently certain toxic effects following the long-continued use of insoluble preparations. This is a rule which applies especially to all insoluble iron preparations, and it is but reasonable to assume that, whatever harm has been done through this means, may have escaped attention, because few physicians are likely to investigate the presence of factitious diseases. Another factor which has contributed to lessen these evils, is the slow process of absorption.

The foregoing observations apply with equal force to the effects of the drug upon the circulatory apparatus. While copper is an active agent in causing contraction of the blood-vessels, iron produces slow contractions, showing that it is less irritant (stimulant) to the nervous system. This may possibly be accounted for on the hypothesis that iron is a normal constituent of the blood. Whether this effect is due to irritation (stimulation) of the vaso-motor nerves, central or paripheral, or to a direct action upon the muscular walls of the blood-vessels, is a question still in doubt. My own impression is, that through the influence of the medicament upon the nerve-cells the large doses, comparatively, arrest their function, when contraction of the muscular structures in the vessels takes place. The ferric salts, owing to their property of coagulating albumin and blood, of course produce more marked effects than the ferrous salts, Digitalis and ergot among the organic, and barium chloride among the inorganic, remedies well-known as vascular tonics, furnish apt illustrations of this important principle.

Iron has a tendency to accumilate in the liver; small doses do not show this tendency, but they may serve to increase the functional activity of this organ, when given in a soluable, non-astringent form, by restoring cell-nutrition to the normal.

The efiect of iron upon muscular structure has long been known to experimental physiologists, but I doubt if this knowledge is appreciated by many practitioners, who regard the possible benefits to be derived from the exhibition of iron preparations in proportion to the amount tolerated by the patient. Now, large doses, while they do not affect the irritability of muscular structure, lessen materially the amount of work it is capable of performing, while small doses increase the capacity of muscle for work. What is most to be desired, therefore, is a preparation not open to the objections inferred from these investigations; but owing to the necessity for consulting the palate of our patients, it is also desirble that the substance should be free from the nauseating effects which are so common to all preparations of iron. The combination, I believe, is to be found in that form known as levulose ferride, which was highly recommended to me several years ago by my friend, Dr. James Collins, of this city.

The preparation known as levulose ferride is one which takes the place or a well-known and popular German product, called *Eizenzucker* (iron sugar), very extensively used in domestic practice. I was led to the employment of iron-sugar on account of its palatability, fastidious patients and children making no objections to it; but this has been supplanted by levulose ferride, which in the form of tablet triturates will be taken as readily as chocolate bon-bons. It is readily soluble in an excess ot water, and practically free from any ferruginous taste or styptic effect when dissolved in the mouth, and is substantially a peptonate. The method of preparing it is briefly as follows: To a certain amount of iron a measured quantity of malt sugar (maltose) is added, and the mixture constantly stirred while exposed on a water-bath. While it possesses all the desirable qualities mentioned, the presence of metalic iron may be determined by chemical analysis, the strength of the product being about three per cent.

This preparation, it will be apparent, will act much less actively as an astringent than even the ferrous preparations; but, of course, it cannot be expected to take the place of the ferric products, which are sometimes demanded, as in the case of intestinal parasites (sarcina ventriculi and lumbricoides). On the other hand it will be especially indicated for the relief of anæmia and chlorosis, owing to its ready absorption, lack of astrigency, and its palatability. In all cases of defective nutrition, from any cause, whether the ingestion of any form of medicament is a trial to the patient, this product will be kindly received. A synopsis of some of the cases in which it is indicated, together with a summary of the effects following its employment may prove interesting to the physician.

During the early summer months, I had under observation a young mother with a six-months old child, who presented a very anæmic condition. I had seen her but once since the delivery of her child, and anticipating that she would not be able to nourish it sufficiently and maintain her health, I had cautioned her in regard to the most appropriate diet. Notwithstadding every care had been used, she was finally compelled to seek medical aid, or go to bed. All that this patient required was something for the purpose of increasing the amount of hæmoglobin, which would restore the integrity of the red corpuscles and improve the oxygen-carrying capacity of the blood. This being most readily accomplished by levulose ferride, she was ordered to take tablets of this preparation, each containing three grains, after meals. To meet the emergency, and increase the patient's strength until such time as the advantages of the iron would be apparent, small doses of strychnine (one-sixtieth grain) were administrated along with the iron. Ordinarily, this class of patients, when they begin in the early summer, suffer more or less from the effects of the heat, and become regular patrons of the doctor; but this patient did not make her appearance again for about two months, when she said she thought it was about time to have a little more of the same medicine. I may mention in passing, that the first medicine was sufficient only to cover the first ten days, and the patient seemed greatly disappointed that she was compelled to return.

So many children are so promptly benefited by the use of a small quantity of iron, that it is a great drawback to us that no palatable preparation has been discovered and put on the market. I have in mind a little fellow, who has long been very much adverse to eating meat, due, I presume, to defective digestion; but for the past few weeks, since he has been taking the levulose ferrid, he seems quite content to eat meat alone, and is becoming strong and robust.

Not long ago I had a visit from a lady, who brought with her a young lad, aged fourteen, who had a most forbidding cadaveric expression, and he could eat no meat. His brother, I was told, had died at about this age from Bright's disease, and this one presented all the symptoms peculiar to the brother who died. Still, with attention to diet, out-door exercise in the country, and a tablet triturate containing three grains of ferride after meals, he made a prompt recovery. Although I was unable to discover any symptoms of Bright's in this instance, I was impressed with the depression due to the anæmic condition; and yet, without some readily assimilable iron preparation, it would have been a tedious process to start him on the way toward recovery.

Late in the spring of the year, a gentleman, aged about thirty-five, called on me, complaining of dyspepsia, although he had been under the treatment of another physician for overwork for four years. After regulating his diet, and adopting treatment calculated to restore the activity of the digestive apparatus, he was placed upon levulose ferride along with strychnine sulphate —three grains of the former in tablet form, one-sixtieth grain of the latter, and did remarkably well on this combination. This product, like all other mild preparations of iron, is mostly indicated in cases of this class, and along with these may be mentioned chorea, convalescence from lingering diseases, like typhoid fever; and in all such instances, I venture to anticipate that the results will be especially favorable where attention is given to dietic measures.

The administration of the remedy may be confined to the use of the powder, which is taken dry on the tongue, dissolved in water or coffee; or it will be found more convenient in the form of tablets, each containing three or five grains. The dose for children ranges from three to ten grains, and for adults from five to thirty grains.

The Levulose Ferrid was obtained through Messrs Eisner & Mendelson Co., of New York, who import this article.

———

The Action of Cod Liver Oil.—Drs. Gautier and Mourgnes, in a recent communication to the Academy of Science discuss at some length the reasons why cod liver oil is superior to other fats as a therapeutic agent, and arrive at the following conclusions:

1. It is more easily assimilated, owing to its containing free fatty acids and some biliary matters which render its emulsion especially easy when it comes in contact with the pancreatic juice.

2. It is rich in phosphates, phosphoric acid, lecithin, and phosphorus in organic combination; the phosphorus, especially in the last mentioned form, is very readily assimilated to form protoplasm, and thus nutrition is very greatly stimulated. The small amount of bromine and iodine being also present as organic compounds, exercise a beneficial effect on the general metabolism.

2. The alkaloids present—butylamine, amylamine, morrhurine—and morrhuic acid stimulate the nervous system, increases the amount of sweat and urine, and acts as nervine tonic.—*Brit. Med. Journal.*

———

Chloroform Narcosis.—After numerous experiments upon animals, Dr. H. Wood has arrived at the following conclusions, in regard to chloroform poisoning:

Avoid the use of all drugs except digitalis and ammonia.

Give the tincture of digitalis hypodermically.

Draw out the tongue and raise the angle of the jaw, and see that respiration is not mechanically impeded.

Invert the patient briefly and temporarily.

Use forced artificial respiration promptly, and in protracted cases apply external warmth and stimulation of the surface by the dry electric brush, etc., and, above all, remember that some at least, and probably deaths which have been set down as due to chloroform and ether, have been produced by alcohol which was given for the relief of the patient.—*Leonard's Med. Journal.*

---

**Condensed Milk for Emulsions.**—It is said that nothing can equal condensed milk for emulsions. To make a 50% emulsion of cod-liver oil:

| | | |
|---|---|---|
| ℞ | Cod-liver oil | 8 fld ozs. |
| | Condensed milk | 3 do |
| | Glycerine or syrup | 3 do |
| | Water | 2 do |

Add of a flavoring oil, such as bitter almond, 10 drops, or wintergreen, 15 drops.

Rub the condensed milk 'round in a dry mortar, and gradually add the cod-liver oil, working, as is customary in making emulsions.

When thoroughly incorporated, add the glycerine and lastly the water and flavoring extract.—*Medical and Surgical Report.*

---

**Sodium Salicylate in Coryza.**—*The Memphis Medical Journal* advises the following in coryza:

| | | |
|---|---|---|
| ℞ | Natr. Salicyl | ʒ iv |
| | Syr. Aur. Cort | ʒ iv |
| | Aqua Menth. Pip | ℥ iv |

M. Sig. A dessert spoon-full every three or four hours.

A dose every three hours until relieved or tinitus aurium results, will control the brow-ache and other unpleasant symptoms.

---

**Dr. Mayham, of Wisconsin,** reports, in the *Medical Standard* a case of pseudocyesis in a woman seventy-two years old, and a mother of several children.

---

**All that can be** positively asserted about the "Lymph" is that it seems to have produced good results in lupus. That sufficient time has not elapsed to judge of its value in lung, brain and joint tuberculosis; that the reaction effects are uncertain, and their diagnostic value is problematical; that the dangers from reaction from the lymph are very great, and have been underestimated. The furore is likely to result in an increased study of pathology and symptomatology of insipient phthisis, and hence cannot fail in the end to benefit science.—*Medical Standard.*

---

**Menthol for Chapped Hands.**—A writer in the *Provincial Mirror Journal* offers the following: Menthol 15 grs., solol ½ drachm, olive oil ½ drachm, lanolin 1½ ounce as a soothing application for chapped hands. The pain, he says, is at once allayed after the first application and the skin is at the same time softened. The fissures will heal promptly under a systematic use of the application once or twice daily.

---

**Infantile Convulsions:**—Dr. Jacobi's usual treatment is to give a purgative dose of calomel, to be followed by four grains of chloral and eight of bromide of potassium; for a child two years old.

---

**Advertising Echoes.**—"Best advertising medium we know; count us permanent patrons."—*Sharpe & Dohme*, (6th year.)

"Renew our contract and continue till forbid."—*W. H. Schieffelin & Co.*

"Will renew for 1891. Contract endorsed." (6th year).—*Park, Davis & Co.*

"Want to thank you for the good our ad. has done."—*Dios Chemical Co.*

"We are well pleased with results, will take a page for another year."—*Feick Bros. & Co.*

"Orders are coming in from all parts of Texas."—*Campho-Phenique.*

"The bulk of our trade in Texas due to an advertisement in your JOURNAL." (Prior to sending our agent.)—*Fairchild Bros. & Foster.*

